# The People Angry

**Published:** 1881-07

**Authors:** 


### THE PEOPLE ANGRY.

MR. ALBERT STICKNEY has just begun, in *Scribner* for July, a short series of three papers on "The People's Problem." In the opening article, which, it is said, is a remarkably able paper, Mr. Stickney says:

"We have just seen a most singular spectacle. The people of the largest and richest city in the country have made a most earnest effort to be allowed to clean their own street with their own money, and to secure their own lives against the dangers of pestilence—and the effort has failed. The reason why it has failed is that the people's own officials, the very men who should have done the work, have made a powerful combination to hinder the work from being done. It has been a battle between the people on the one side and the men who should be their honest servants on the other side—and the servants have won the battle. Singularly, too, in this matter, which has been on the part of the citizens nothing but an effort to save life, we have seen our public officials, of the national, State, and city governments, all combined together to resist this effort of the people of one city.

"We have substantially the same state of things in nearly every large centre of wealth and population in the country—public officials are banded together to draw money from the public treasury instead of doing well the public work. We have the same condition of things in our State legislature. The people's work is not well done. The people's offices are not used for the people's purposes. And at the national capital, where we ought to have a body of public servants watching over the nation's welfare, the people's representatives are wasting their time in a mere struggle for place. Great national questions need to be wisely handled by the men who control our national affairs. But the men who are highest in the nation's service have brought the business of the people of the United States to a standstill, while they wrangle over the appointment of doorkeepers and revenue officials.

"The people are angry. They do well to be angry; but they are angry at the wrong thing. They are angry at certain men; they should be angry at the system, which has made the men what they are.

"Our political machinery has a radical, fundamental fault. The material which we use in our public service is the same that we use in all private industrial enterprises—men—human nature. In private life the material serves its uses most nobly. Yet in our public affairs we have become so accustomed to downright robbery at the hands of men holding public place, that the whole community gives a sigh of relief when Congress or a State legislature adjourns, at the thought that their power for evil is for a time ended. Yet most of these men who fill our public offices are, in private life, honest men.

"But to say that there is some fault in our political machinery avails nothing. We must go further than that, and find precisely and accurately what the fault is. To find out what the precise fault is, if we stop there, will avail little. We must find what is the remedy. And even to find the precise remedy is not enough. We must go further, and convince men that the remedy can be applied—must show *how* it can be applied."

